# Effects of baicalein on IL-1β-induced inflammation and apoptosis in rat articular chondrocytes

**DOI:** 10.18632/oncotarget.21796

**Published:** 2017-10-11

**Authors:** Yue Li, Jinglu Wang, Xiaopeng Song, Hui Bai, Tianwen Ma, Zhiheng Zhang, Xinran Li, Renli Jiang, Guanying Wang, Xiaojing Fan, Xu Liu, Li Gao

**Affiliations:** ^1^ College of Veterinary Medicine, Northeast Agricultural University, Harbin, Heilongjiang, People’s Republic of China

**Keywords:** baicalein, osteoarthritis, chondrocyte, NF-κB, apoptosis, Immunology and Microbiology Section, Immune response, Immunity

## Abstract

In osteoarthritis (OA), activated synoviocytes and articular chondrocytes produce pro-inflammatory cytokines, such as IL-1β, that promote chondrocyte apoptosis and activate the NF-κB signaling pathway to induce catabolic factors. In this study, we examined the anti-inflammatory and anti-apoptotic effect of baicalein on IL-1β signaling and NF-κB-regulated gene products in rat chondrocytes. Rat chondrocytes were pretreated with 10 ng/ml IL-1β for 24 h and then co-treated with 10 ng/ml IL-1β and 50 μM baicalein for 0, 12, 24, 36 and 48h. The expression levels of poly(ADP-ribose) polymerase (PARP), Bcl-2, caspase-3, matrix metalloproteinase (MMP)-9, MMP-3, cyclooxygenase (COX)-2 and SOX-9 were detected by Western blot and quantitative reverse transcription-PCR (qPCR). The effects of baicalein on the translocation and phosphorylation of the NF-κB system were studied by Western blotting and immunofluorescence. Baicalein stimulated the expression of anti-apoptotic genes and reduced the pro-apoptotic and pro-inflammatory gene products in chondrocytes. Baicalein promoted SOX-9 expression in a time-dependent manner in chondrocytes. Baicalein inhibited the NF-κB activation that was induced by IL-1β in a time-dependent manner in chondrocytes. Our results suggest that the anti-inflammatory and anti-apoptotic effects of baicalein are mediated through the inhibition of the translocation of phosphorylated p65 to the nucleus.

## INTRODUCTION

Osteoarthritis (OA) is a common arthritic disease that gradually leads to cellular changes, structural defects and dysfunction of all the joint compartments [1]. This “whole joint disorder” is characterized by the breakdown of articular cartilage, subchondral bone sclerosis, osteophyte formation, inflammation of the synovial membrane and vascularization of the articular cartilage [2, 3]. The causes and origins of OA have not been fully elucidated. Nevertheless, pro-inflammatory cytokines, such as IL-1β, are produced by activated synoviocytes and articular chondrocytes and play a pivotal role in the pathogenesis of OA [4].

Baicalein is a flavonoid extracted from the dry root of *Scutellaria* (Huang Qin in Chinese), which is widely used in China and several other countries [5]. Baicalein is the main active constituent in a variety of flavones, amino acids and essential oils [5]. The traditional Chinese herb extract has shown antioxidant [6], anti-viral [7-11], anti-thrombotic [12, 13], anti-inflammatory [14], anti-cardiovascular illness [13, 15] and anti-tumor effects *in vitro* [16, 17] and *in vivo* [18-20].

Currently, one study shows that nuclear factor-κB (NF-κB) activity was reduced in myeloma cell lines after baicalein treatment [21]. NF-κB is an inducible transcription factor that controls the expression of more than one hundred genes involved in immunity, inflammation, proliferation, and defense against apoptosis [22]. Another report shows that baicalein lowered the levels of matrix metalloproteinase (MMP)-2 and MMP-9 to inhibit tumor cell metastasis [23]. Another report reveals that *Scutellaria baicalensis* extracts inhibit cyclooxygenase (COX)-2 expression *in vitro* [19]. Apoptosis was found to correlate with cartilage destruction and matrix depletion in human osteoarthritic tissue specimens [24]. Chondrocytes isolated from OA cartilage, but not those isolated from normal donors, exhibited morphological evidence of apoptosis [25, 26]. These findings suggest that baicalein has the potential to inhibit apoptosis and MMPs expression in OA treatment.

In this study, we examined the anti-inflammatory and anti-apoptotic effect of baicalein on IL-1β signaling and NF-κB-regulated gene products in rat chondrocytes.

## RESULTS

### Effects of baicalein on chondrocyte viability

**Figure 1:**
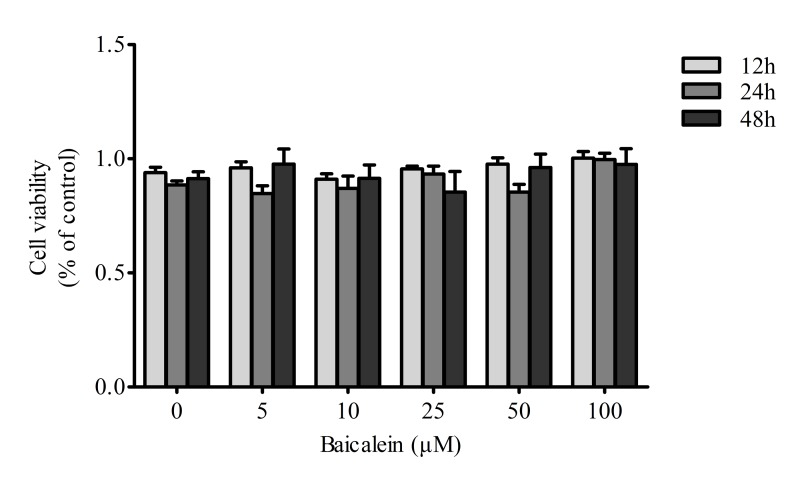
Effects of baicalein on the viability of chondrocytes Rat chondrocytes were treated with 0, 5, 10, 25, 50 and 100 μM baicalein for 12, 24 and 48 h and evaluated by CCK-8 assay. Values are shown as the means ± SEM (*n* = 6). * *P* < 0.05; ** *P* < 0.01;*** *P* < 0.001.

In the present study, we evaluated the effects of baicalein on chondrocyte viability. Proliferation and viability assays performed with the CCK-8 kit indicated that baicalein showed no side-effects on cell viability (*P* > 0.05), and the viability was not recovered in a dose-dependent manner by treatment with baicalein. (Figure 1)

### Baicalein stimulates the expression of anti-apoptotic genes and inhibits pro-apoptotic gene products in chondrocytes

**Figure 2:**
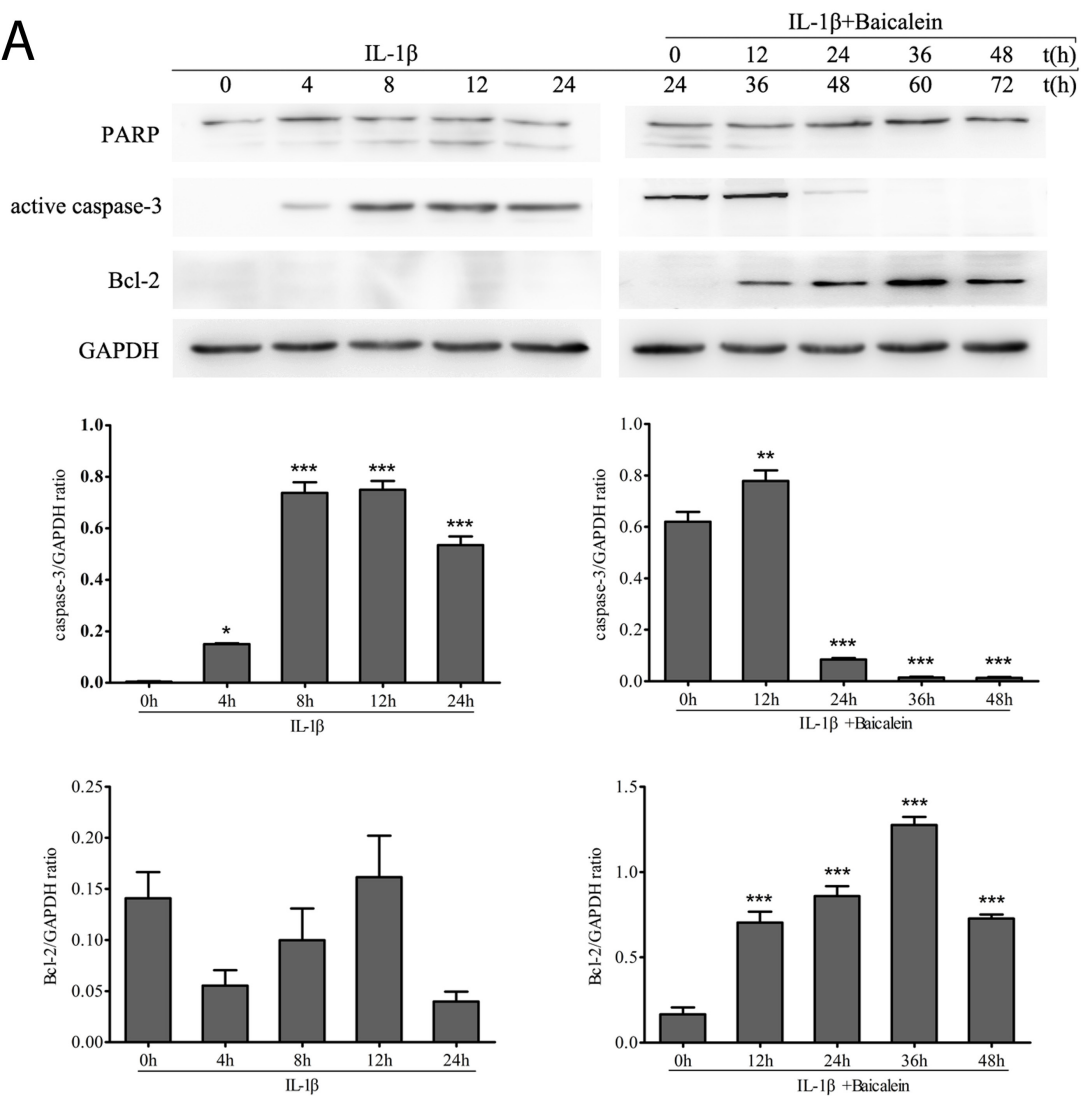

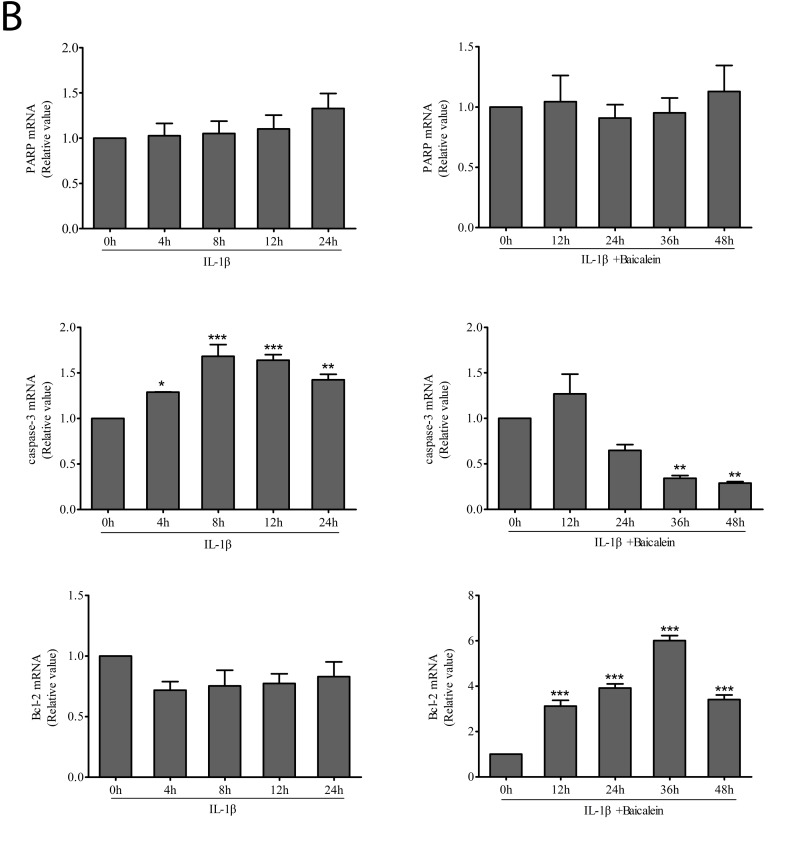
Baicalein stimulates the expression of anti-apoptotic proteins and inhibits pro-apoptotic gene products in chondrocytes **A.**, **B.** Protein expression **A.** of PARP, active caspase-3 and Bcl-2; and their gene expression **B.** in chondrocytes, as estimated by Western blot and qPCR. Cultures were treated with 10 ng/ml IL-1β for 0, 4, 8, 12 and 24 h or co-treated with 50 μM baicalein and 10 ng/ml IL-1β for 0, 12, 24, 36 and 48 h after exposure to 10 ng/ml IL-1β alone for 24 h. Values are shown as the means ± SEM (*n* = 5). * *P* < 0.05;** *P* < 0.01;*** *P* < 0.001.

Western blot analysis was performed with antibodies against the DNA repair enzyme PARP because cell degeneration and apoptosis are marked by enhanced caspase-mediated PARP cleavage. Treatment with baicalein inhibited IL-1β-induced PARP cleavage, and the levels were similar to those in control cultures. (Figure 2)

Furthermore, we wanted to know whether baicalein also suppressed the IL-1β-induced pro-apoptotic gene product and activated caspase-3 in the same cell cultures. Therefore, chondrocytes were incubated with IL-1β (10 ng/ml) alone for 0, 4, 8, 12 and 24 h or co-incubated with 10 ng/ml IL-1β and 50 μM baicalein for 0, 12, 24, 36 and 48 h after stimulation with 10 ng/ml IL-1β for 24 h. As shown in Figure 2, co-treatment with 10 ng/ml IL-1β and 50 μM baicalein significantly down-regulated the level of biologically active caspase-3 in IL-1β-stimulated cultures relative to the level in chondrocytes stimulated with IL-1β alone.

NF-κB is known to regulate the expression of the anti-apoptotic protein Bcl-2 [27, 28]. To evaluate whether baicalein could modulate the expression of this anti-apoptotic gene product, serum-starved human articular chondrocytes were stimulated with 10 ng/ml IL-1β alone for 0, 4, 8, 12 and 24 h or co-treated with 10 ng/ml IL-1β and 50 μM baicalein for 0, 12, 24, 36 and 48 h after stimulation with 10 ng/ml IL-1β for 24 h. Then, whole cell extracts were prepared and analyzed by Western blot and qPCR. As shown in Figure 2, IL-1β not stimulated the expression of Bcl-2. In contrast, the combined treatment of 10 ng/ml IL-1β and 50 μM baicalein stimulated the expression of Bcl-2 in chondrocytes. Taken together, these results indicate that baicalein suppressed IL-1β-induced apoptosis in chondrocytes.

### Baicalein inhibits IL-1β-induced NF-κB-dependent pro-inflammatory gene products in chondrocytes

**Figure 3:**
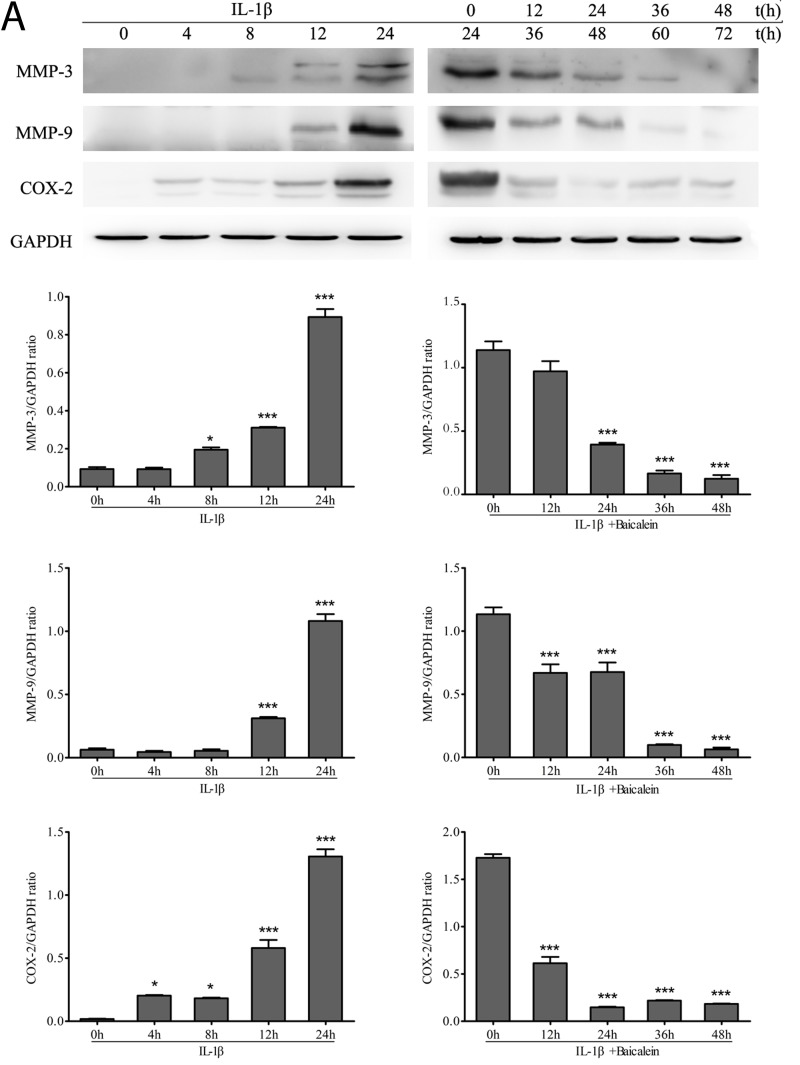

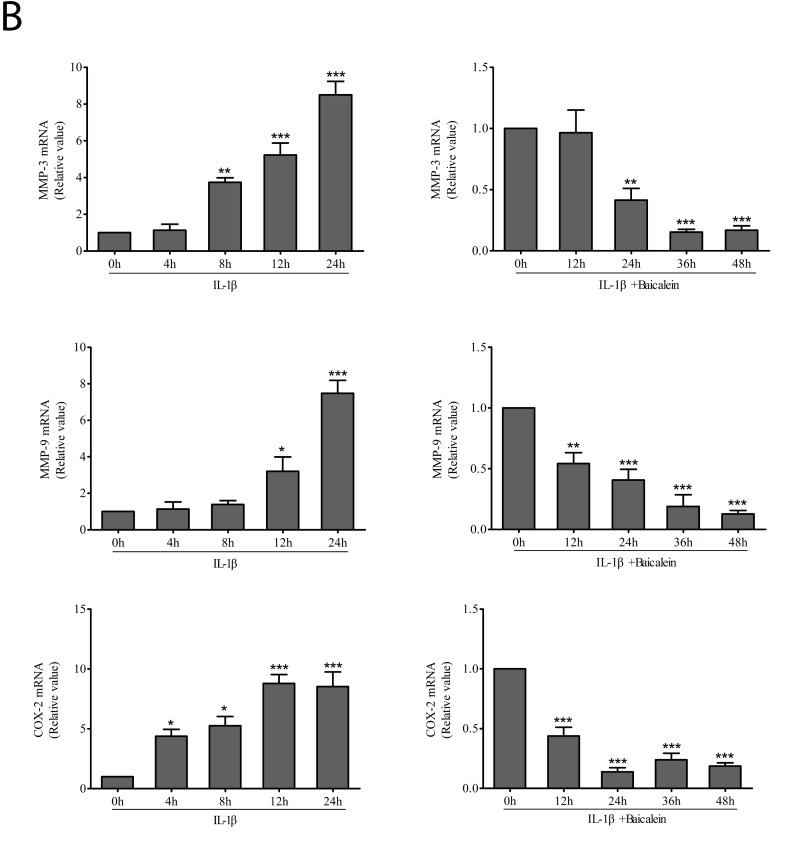
Up-regulation of pro-inflammatory enzymes by IL-1β in chondrocytes is inhibited by baicalein **A.**, **B.** Protein expression **A.** of MMP-3, MMP-9 and COX-2; and their gene expression **B.** in chondrocytes, as estimated by Western blot and qPCR. Cells were treated as described in Figure 2. Values are shown as the means ± SEM (*n* = 5). * *P* < 0.05;** *P* < 0.01;*** *P* < 0.001.

We investigated whether baicalein could modulate IL-1β-induced NF-κB-regulated gene products involved in the inflammation of chondrocytes. It has been shown previously in chondrocytes that IL-1β stimulation activates COX-2 [29], MMPs [30] expression. We therefore investigated whether this natural product was able to inhibit the IL-1β-induced expression of these proteins. Serum-starved human articular chondrocytes were treated with 10 ng/ml IL-1β for 24 h and then co-treated with 50 μM baicalein and 10 ng/ml IL-1β for 0, 12, 24, 36 and 48 h, and total cellular protein and mRNA were prepared and analyzed by Western blot and qPCR. IL-1β induced the expression of COX-2, MMP-3 and MMP-9 in a time-dependent manner, and the combined treatment of baicalein and IL-1β inhibited the expression of the above-mentioned proteins in chondrocytes (Figure 3).

### Effect of baicalein on SOX-9 in chondrocytes

**Figure 4:**
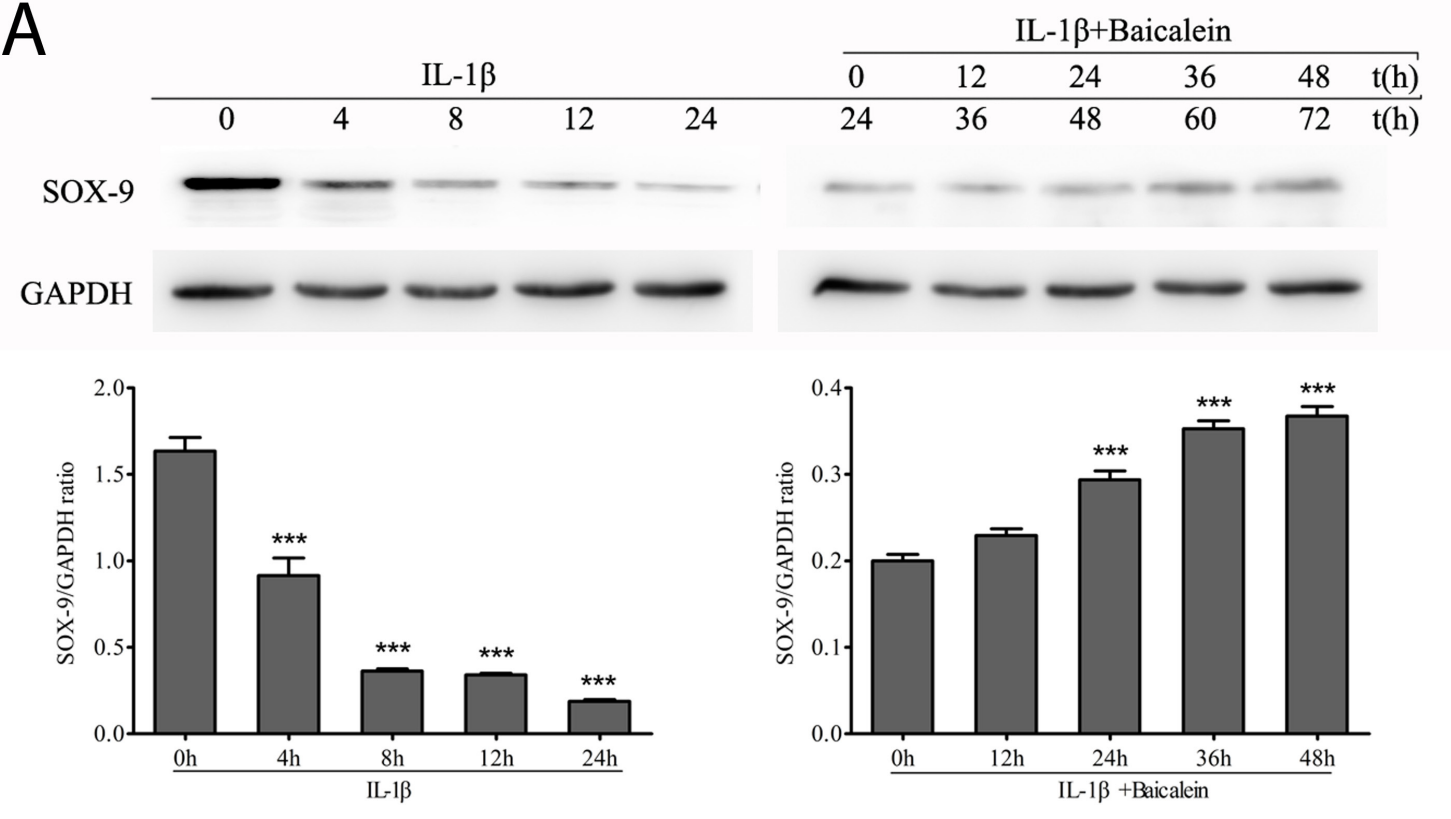

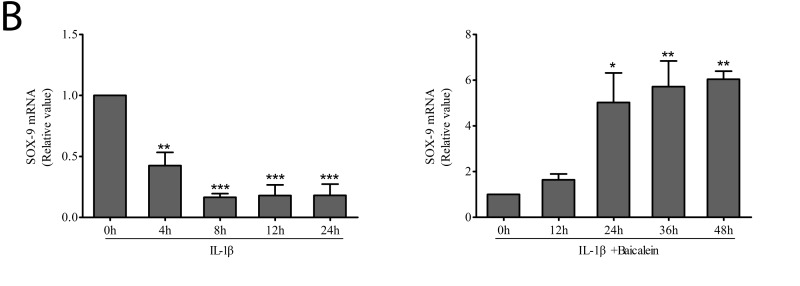
Effects of baicalein on IL-1β-induced inhibition of SOX-9 production in chondrocytes **A.**, **B.** Protein expression **A.** of SOX-9 and their gene expression **B.** in chondrocytes, as estimated by Western blot and qPCR. Cells were treated as described in Figure 2. Values are shown as the means ± SEM (*n* = 5). * *P* < 0.05;** *P* < 0.01;*** *P* < 0.001.

SOX-9 is a master specific transcription factor that controls the expression of chondrocyte-specific ECM protein genes and plays a pivotal role in chondrocyte differentiation [31]. To test the hypothesis whether the phytochemical is able to activate the transcription factor SOX-9 in chondrocytes, monolayer cultures of chondrocytes were stimulated with 50 μM baicalein and 10 ng/ml IL-1β for 0, 12, 24, 36 and 48 h after incubated with 10 ng/ml IL-1β alone for 24 h, and cell lysates were analyzed by Western blot and qPCR. The results demonstrated that baicalein-induced SOX-9 increasing is stronger than the IL-1β-induced SOX-9 decreasing (Figure 4).

### Baicalein inhibits NF-κB activation induced by IL-1β in a time-dependent manner in chondrocytes

#### Effect of baicalein on IL-1β-induced phosphorylation of NF-κB in the cytoplasm

**Figure 5:**
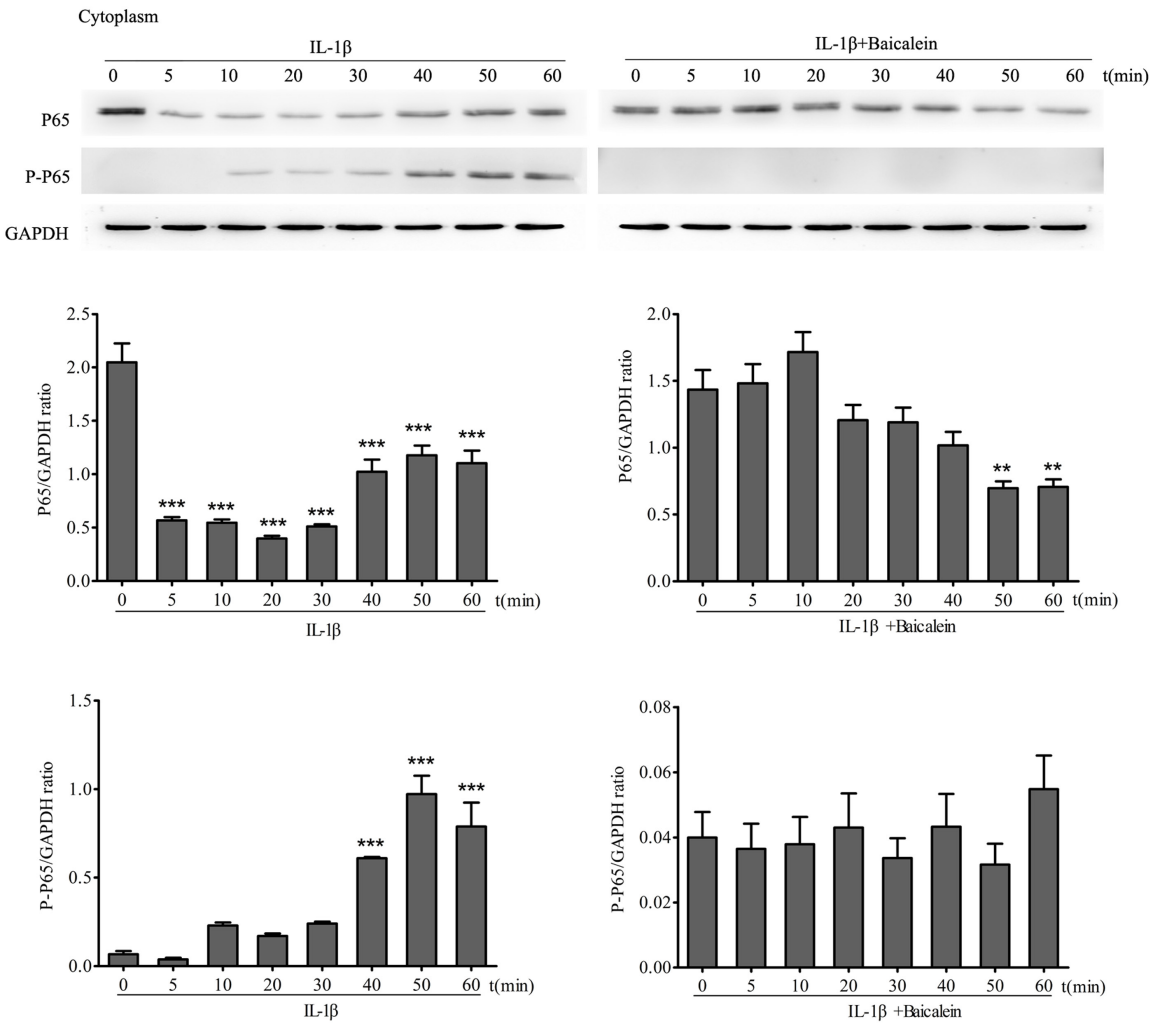
Baicalein inhibits IL-1β-induced phosphorylation and translocation of p65 in cytoplasmic extracts of chondrocytes Western blot analysis with IL-1β-treated cytoplasmic extract. Serum-starved chondrocytes (0.1×10^6^ cells/ml) were treated with 10 ng/ml IL-1β for 0, 5, 10, 20, 30, 40, 50 and 60 min. Other cultures were initially treated with 10 ng/ml IL-1β for identical time periods and then co-treated with 50 μM baicalein for 4 h. Values are shown as the means ± SEM (*n* = 5). * *P* < 0.05;** *P* < 0.01;*** *P* < 0.001.

To test the effect of baicalein on IL-1β activation of NF-κB, serum-starved chondrocytes were treated with 10 ng/ml IL-1β for 0, 10, 15, 30 and 60 min alone, and other cultures were treated with 10 ng/ml IL-1β for the same time periods alone and together with 50 μM baicalein for 4 h. Cytoplasmic extracts were investigated for the expression of pan/ phospho p65 by Western blot analysis. p65 phosphorylation is a known prerequisite for NF-κB transcriptional functions, and this phosphorylation is mediated by IKK [32]. As shown in Figure 5, IL-1β induced phosphorylation of the p65 cytoplasmic pool in a time-dependent manner. This phosphorylation could be observed as early as 10 min and increased up to 50 min. In chondrocytes that were treated with baicalein, the IL-1β-induced activation of cytoplasmic p65 was completely blocked.

#### Effects of baicalein on IL-1β-induced phosphorylation of p65 in the nucleus

**Figure 6:**
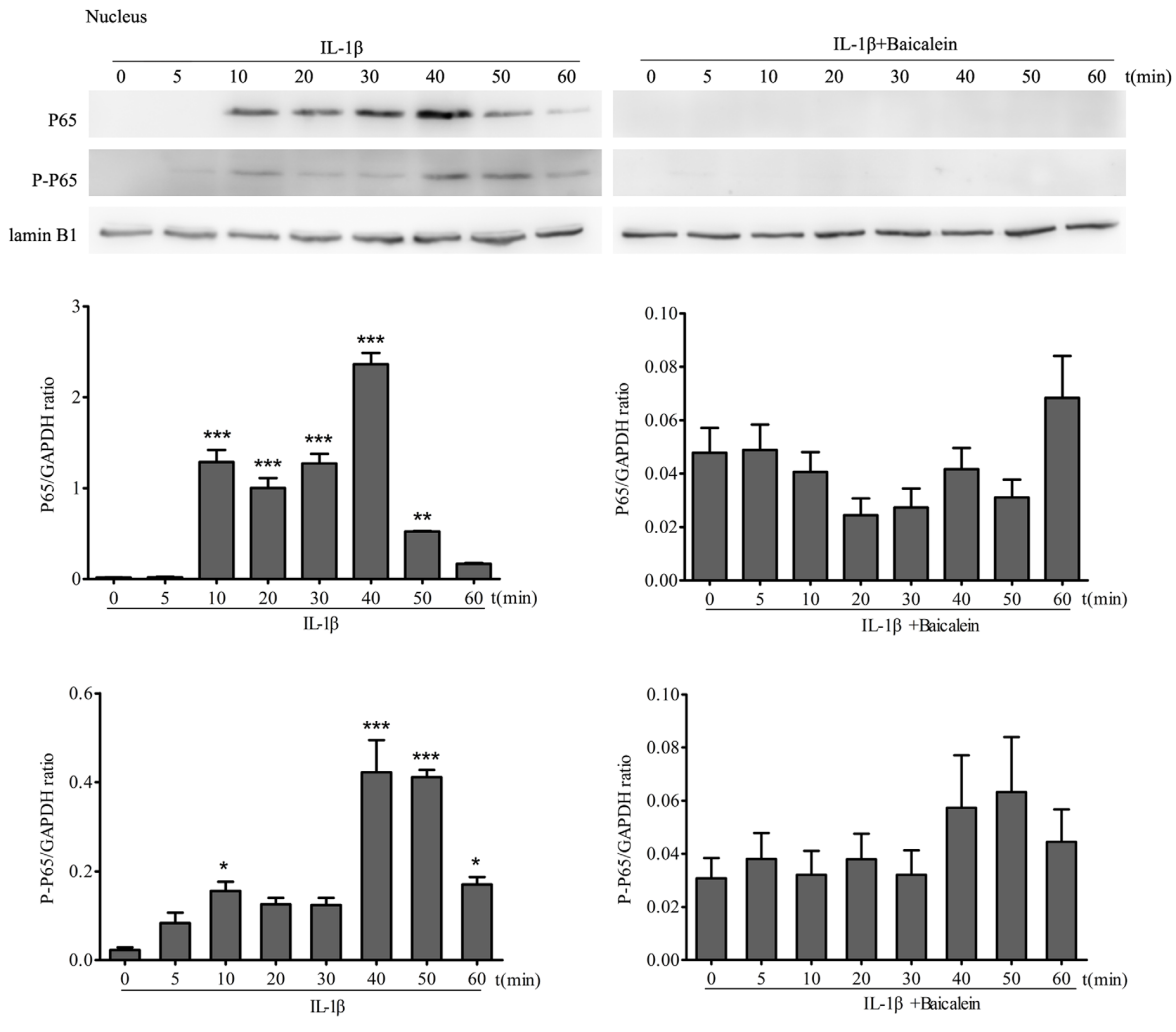
Baicalein inhibits IL-1β-induced phosphorylation and translocation of p65 in nuclear extracts of chondrocytes Western blot analysis with IL-1β-treated nuclear extract. Cells were treated as described in Figure 5. Values are shown as the means ± SEM (*n* = 5). * *P* < 0.05;** *P* < 0.01;*** *P* < 0.001.

Translocation of NF-κB to the nucleus is necessary for the regulation of gene expression. The translocation of activated NF-κB is preceded by phosphorylation of the p65 subunit of NF-κB [33]. Therefore, to test this, protein extracts of serum-starved chondrocyte nuclear extracts were probed for the pan and phosphorylated p65 NF-κB-subunit after stimulation of chondrocytes with 10 ng/ml IL-1β for 0, 10, 15, 30 and 60 min. To show the effect of baicalein on p65 phosphorylation, other cultures were first treated with 10 ng/ml IL-1β alone for the same time periods, and then every time course was co-treated with 50 μM baicalein for 4 h. Chondrocytes treated with 10 ng/ml IL-1β at different time points revealed a visible increase in pan p65 subunit and phospho p65 in a time-dependent manner in the nuclear extracts (Figure 6). The co-treatment of chondrocytes with baicalein and IL-1β abolished the pan p65 subunit and the IL-1β-dependent phosphorylation of p65 in a time-dependent manner in the nucleus. These results clearly show that baicalein inhibits the IL-1β-induced translocation of p65 to the nucleus. The synthesis of the Lamin B1 protein remained unaffected.

#### Baicalein blocks IL-1β-induced nuclear translocation of NF-κB visualized by immunofluorescence microscopy

**Figure 7:**
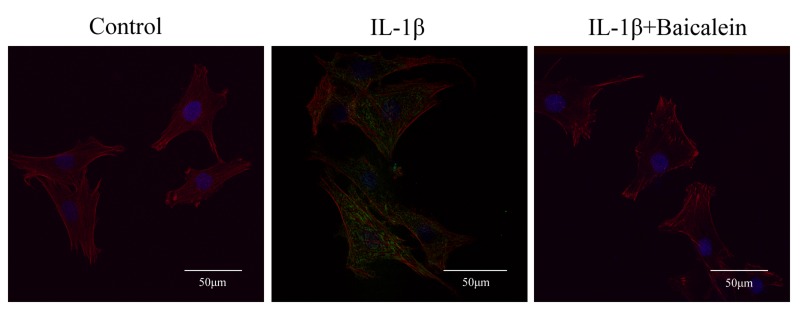
Baicalein inhibits IL-1β-induced nuclear translocation of phospho p65 as revealed by immunofluorescence microscopy Chondrocyte cultures either served as controls or were treated with IL-1β alone for 30 min or co-treated with 10 ng/ml IL-1β and 50 μM baicalein for 2 h after treatment with IL-1β alone for 30 min. Confocal immunofluorescent analysis of chondrocytes using Phospho-NF-κB p65 Antibody (green), Phalloidin (red) and DAPI (blue).

Immunofluorescence microscopy was employed to reveal translocation of the phosphorylated p65 subunit of NF-κB from the chondrocyte cytoplasm to the nucleus in response to NF-κB activation by IL-1β. Unstimulated chondrocytes or those stimulated with 10 ng/ml IL-1β alone for 30 min or co-treated with 10 ng/ml IL-1β for 30 min and then 50 μM baicalein for 2 h before immunolabeling with anti-phospho p65 antibody were compared. The control chondrocytes showed only cytoplasmic labeling of phospho p65. IL-1β-stimulated cells revealed clear and intense cytoplasmic and nuclear staining for phospho p65. Co-treatment of chondrocytes with IL-1β and baicalein resulted in inhibition of nuclear translocation of activated phospho p65, decreased cytoplasmic staining for this protein and decreased activation of NF-κB (Figure 7). These immunomorphological findings were consistent with the NF-κB inhibition observed by Western blot.

## DISCUSSION

This study led to the following findings: (1) baicalein shows no side-effects on chondrocyte viability, and viability was not recovered in a dose-dependent manner by treatment with baicalein; (2) IL-1β-stimulated cells treated with baicalein inhibit the activation of PARP cleavage; (3) baicalein exerts anti-inflammatory and anti-apoptotic effects; (4) baicalein suppresses the IL-1β-induced down-regulation of the cartilage-specific master transcription factor SOX-9; (5) baicalein antagonizes the IL-1β-induced phosphorylation and nuclear translocation of the p65 NF-kB subunit.

Baicalein (5, 6, 7-trihydroxyflavone), the principal component of the roots of *Scutellaria baicalensis* Georgi extract, is known as Huang Qin in Chinese traditional medicine [34]. Due to its excellent biological action, baicalein has been the focus of pharmaceutical, cosmetic, and food industry studies. It has been reported that the herbal extract shows anti-inflammatory effects and improvements of mitochondrial dysfunction [35]. In addition, pre-incubation of baicalein caused an increase of Bcl-2 [36] and blocked the activation of caspases [37]. The expression of caspase-3, the cellular death marker, and Bcl-2, the anti-apoptotic member of the Bcl-2 family, was shown to be suppressed by NF-κB [38-40]. In the present study, we found that baicalein inhibited the expression of caspase-3 in IL-1β-stimulated chondrocytes and promoted the expression of Bcl-2 in the same condition. It is well known that poly (ADP-ribose) polymerase (PARP) is a nuclear enzyme that can be activated in cells experiencing stress and/or DNA damage and is cleaved by the caspase family *in vitro*. We also found that baicalein antagonized the cleavage of PARP in the chondrocytes incubated with IL-1β.

The pro-inflammatory mediators IL-1β and tumor necrosis factor-α (TNF-α), which are produced by synovitis, activate chondrocytes and cause phenotypic shifts, apoptosis and aberrant expression of inflammation-related genes, including COX-2 and MMPs [41-43], which play key roles in the initiation and perpetuation of osteoarthritic cartilage destruction [44, 45]. Seven matrix metalloproteinases have been found during a variety of circumstances in articular cartilage, including MMP-1, MMP-2, MMP-3, MMP-8, MMP-9, MMP-13 and MMP-14 [46]. The expression of MMP-3 and MMP-9 in cartilage is characteristic of the pathologic circumstances of OA. MMP-3 degrades a wide array of extracellular molecules, such as collagen type II and various proteoglycans, and up-regulates the expression of other MMPs [47]. MMP-9 reveals extremely strong activity in the disintegration of all collagen types but also native collagens, like type IV and type XI [45]. In this study, IL-1β stimulated the time-dependent up-regulation of MMPs, while baicalein showed an antagonistic effect. COX-2 is an important mediator of pain and inflammation in osteoarthritic joints [48]. COX-2, but not COX-1, production is stimulated by IL-1β and TNF-α [29, 49]. COX-2 activity leads to PGE2 and thromboxane production [50-52]. PGE2 exerts some additional catabolic effects on chondrocytes, such as decreased proliferation of chondrocytes and inhibition of proteoglycan synthesis [47]. As a result, baicalein suppressed the synthesis of COX-2 in IL-1β-stimulated chondrocytes, but the expression level of COX-2 remained higher than that in un-stimulated chondrocytes. We propose that the anti-inflammatory action and anti-apoptotic effects of baicalein are due to the activation of NF-κB signaling because the activation of canonical NF-κB signaling is required for the expression of MMPs and COX-2 [53].

Activation of NF-κB is crucial to the pathogenesis of arthritis, and targeting NF-κB both alleviates inflammation and inhibits hyperplasia in arthritis [54]. Thus, NF-κB represents an essential target for the treatment of OA. In the inactive state, transcription factors of the NF-κB family are held in check by inhibitors called inhibitor of κB (IκBs) in the cytoplasm [55]. Upon stimulation by different stressors, the phosphorylated p65 subunit of NF-κB translocates to the nucleus and binds DNA [56]. In addition, reduced NF-κB activity was observed in myeloma cell lines after baicalein treatment [21]. In the present study, the phosphorylation of p65 in nuclear extracts of chondrocytes cultured with IL-1β was significantly increased, as demonstrated by Western blot analysis, implying that phospho p65 had translocated to the nucleus from the cytoplasm, thus activating NF-κB. The same phenomenon could also be demonstrated by immunofluorescence microscopy. Baicalein inhibited the translocation of phosphorylated p65 to the nucleus, which was confirmed by Western blot analysis and immunofluorescence microscopy.

SOX-9 is essential for chondrogenesis and chondrocyte differentiation [57-59]. It is unclear whether the differentiation state of chondrocytes could cause OA [60]. However, a recent study showed that the loss of phenotypic stability of articular chondrocytes could indeed constitute an initiating event [61]. SOX-9 expression is essential for the survival of chondrocytes, as it can progress chondrocytes to hypertrophy [58, 59, 62]. SOX9 has been shown to both activate and inhibit the expression of type X collagen in hypertrophic chondrocytes [63, 64]. In this study, we observed that baicalein promoted the expression of SOX-9 that was reduced in chondrocytes after IL-1β treatment, though the quantity was less than in normal chondrocytes. Therefore, more studies are needed to obtain a better understanding of the *in vivo* effects of baicalein on cartilage damage in OA.

In conclusion, these results suggest that the anti-inflammatory and anti-apoptotic effects of baicalein are mediated through inhibition of the translocation of phosphorylated p65 to the nucleus. While a large variety of intracellular signaling pathways are involved in OA, it is unclear whether there are additional molecular targets of baicalein. Thus, further *in vitro* and *in vivo* studies will be required to explore the potential effects of baicalein for the prevention and treatment of OA.

## MATERIALS AND METHODS

### Antibodies

Monoclonal anti-SOX-9 and anti-MMP-9 (EP1255Y) were obtained from Abcam (Cambridge Science Park, UK). Antibodies raised against PARP (46D11), Bcl-2 (50E3), cleaved caspase-3 (8G10), MMP-3 (D7F5B), COX-2 (D5H5), NF-κB p65 (D14E12), phospho-NF-κB p65 (Ser536, 93H1) and Anti-rabbit IgG (H+L), F(ab’)2 Fragment (Alexa Fluor^®^ 488 Conjugate) were obtained from Cell Signaling Technology (Beverly, MA, USA). Antibodies to GAPDH, sheep anti-mouse and sheep anti-rabbit secondary antibodies were obtained from ZSGB-BIO (Beijing, China). All antibodies were used at concentrations and dilutions recommended by the manufacturer (dilutions ranged from 1:100 for immunomorphological experiments to 1:10,00 for Western blot analysis).

### Growth medium and chemicals

Growth medium (DMEM/F-12 (50/50) containing 10% FBS, 50 μg/ml streptomycin, 50 IU/ml penicillin, essential amino acids and L-glutamine) was obtained from Gibco (Life Technologies, NY, USA). Baicalein was purchased from Sigma-Aldrich (St. Louis, MO, USA). Baicalein was prepared as a 100 μM solution in dimethylsulfoxide (DMSO) and then further diluted in cell culture medium. IL-1β was obtained from PeproTech (NJ, USA). Cell lysis buffer for Western blot was ordered from Beyotime (Jiangsu, China). Cell counting kit-8 (CCK-8) was acquired from Dojindo (Kyushu, Japan). RNAiso Plus, PrimeScript RT reagent kit and SYBR Premix Ex Taq was obtained from Takara (Beijing, China). Alexa Fluor^®^ 555 Phalloidin and DAPI were purchased from Cell Signaling Technology (Beverly, MA, USA).

### Chondrocyte isolation and culture

Primary cultures of rat chondrocytes were isolated from articular cartilage as previously described [65]. Cartilage slices were digested primarily with 0.25% (v/v) trypsin for 30 min at 37°C and subsequently with 0.2% (w/v) collagenase Type II for 4-8 hours at 37°C in a shaker. Primary chondrocytes were cultured at a density of 2×10^6^ cells/ml in growth medium at 37°C with 5% CO_2_. When the cells reached 80% confluence, they were passaged, and the chondrocytes of passage 2 were used for subsequent experiments.

### Cell viability assay

Cell viability was measured by colorimetric CCK-8 assay. Briefly, 5,000 chondrocytes per well were cultured for 24 h in a 96-well-plate and then washed three times with serum-starved medium and incubated for 12 h with serum-starved medium (0.5% FBS). Serum-starved human articular chondrocytes were treated with 0 μM, 5 μM, 10 μM, 25 μM, 50 μM or 100 μM baicalein for 12 h, 24 h or 48 h at 37°C. CCK-8 was added to the cells and incubated for 4 h at 37°C. The absorbance was read at 450 nm using a microplate reader (BioRad, CA, USA).

### Experimental design

Serum-starved articular chondrocytes were treated with 10 ng/ml IL-1β alone for 0, 4, 8, 12 and 24 h or pretreated with 10 ng/ml IL-1β for 24 h followed by co-treatment with 10 ng/ml IL-1β and 50 μM baicalein for 0, 12, 24, 36 and 48 h. The experiments described in the present study were specifically designed to mimic the cellular events that occur in the clinical condition of OA.

For the investigation of NF-κB translocation and phosphorylation, serum-starved chondrocyte cultures were treated either with 10 ng/ml IL-1β for 0, 5, 10, 20, 30, 40, 50 and 60 min or pretreated with 10 ng/ml IL-1β for 0, 5, 10, 20, 30, 40, 50 and 60 min and then co-treated with 10 ng/ml IL-1β and 50 μM baicalein for 4 h.

### Immunofluorescence microscopy

Serum-starved chondrocytes were either left untreated, treated with 10 ng/ml IL-1β alone for 30 min or pretreated with 10 ng/ml IL-1β for 30 min followed by co-treatment with 10 ng/ml IL-1β and 50 μM baicalein for 2 h. Samples were rinsed three times in phosphate buffered saline (PBS) and then overlaid with protease-free bovine serum albumin (BSA) and 0.3% Triton-X100 for 60 min at RT, rinsed with PBS and incubated with primary antibodies (phospho-p65, 1:100 in PBS) in a humidified chamber overnight at 4°C. They were then gently washed three times with PBS before incubation with secondary antibody (goat anti-rabbit immunoglobulin, 1:250 in PBS) for 90 min at RT. After rinsing with PBS three times, the samples were incubated with DAPI for 10 min at RT. Cells were washed three times with PBS and then incubated with Phalloidin for 15 min at RT. Finally, the samples were rinsed three times with PBS before being covered with water-soluble mounting medium and examined under laser confocal fluorescence microscopy.

### Western blot analysis

Total cellular protein, nuclear or cytoplasmic fractions in articular chondrocytes were extracted with cell lysis buffer, and the concentrations were then determined according to the bicinchoninic acid system using BSA as a standard. Equal quantities of total proteins were separated by SDS-PAGE and then transferred onto nitrocellulose filter (NC) membranes. The membrane was blocked with 5% (w/v) skimmed milk powder or 3% (w/v) BSA at RT for 1 h and incubated with primary antibodies at 4°C overnight. After rinsing three times in Tris-buffered saline (TBS) with 20% (v/v) Tween 20, the membrane was incubated with the secondary antibody conjugated with horseradish peroxidase at room temperature for 1 h. Finally, the blots were developed with the ECL reagent through Western Blotting Detection System (Amersham Life Science, UK).

### Quantitative reverse transcription-PCR (qPCR)

Total RNA was isolated from different samples using RNAiso Plus according to the manufacturer’s instructions. 500 ng of total RNA was reverse-transcribed using the PrimeScript RT reagent kit. The levels of mRNA expression were determined using SYBR Premix Ex Taq. Expression of GAPDH was used as endogenous control. Quantitative realtime PCR data were calculated by the 2^(-ΔΔCT)^ method. Primer sequences are listed in Table 1.

**Table 1:** Primer sequences used in quantitative reverse transcription-PCR.

Gene name		Primer sequences (5’ to 3’)
*PARP*	Forward	TGCAGAGTGTTCCAGACCAG
	Reverse	CACCCTCCAAGAAGAGCAAG
*active caspase-3*	Forward	GCTGGACTGCGGTATTGAGA
	Reverse	TAACCGGGTGCGGTAGAGTA
*Bcl-2*	Forward	GGATGACTTCTCTCGTCGCT
	Reverse	GACATCTCCCTGTTGACGCT
*MMP-3*	Forward	TTTGGCCGTCTCTTCCATCC
	Reverse	GCATCGATCTTCTGGACGGT
*MMP-9*	Forward	GATCCCCAGAGCGTTACTCG
	Reverse	GTTGTGGAAACTCACACGCC
*COX-2*	Forward	CAACACCTGAGCGGTTACCA
	Reverse	CAGCGGATGCCAGTGATAGA
*SOX-9*	Forward	GCGACGTCATCTCCAACATC
	Reverse	ATGCCGTAGCTGCCAGTGTA
*GAPDH*	Forward	GATGCCCCCATGTTTGTGAT
	Reverse	GGCATGGACTGTGGTCATGAG

### Statistical analysis

All experiments were performed in triplicate. The statistical analysis was performed using GraphPad Prism 7.0 (GraphPad Software Inc., USA) software. One-way analysis of variance (ANOVA) analysis was used for the statistical comparison of multiple groups. Values were expressed as the means ± standard error of mean (SEM). *P* < 0.05 was considered significant.
